# Mesoscale Variation of Mechanisms Contributing to Stability in Rocky Shore Communities

**DOI:** 10.1371/journal.pone.0054159

**Published:** 2013-01-11

**Authors:** Nelson Valdivia, Andrés E. González, Tatiana Manzur, Bernardo R. Broitman

**Affiliations:** 1 Universidad Austral de Chile, Instituto de Ciencias Marinas y Limnológicas, Laboratorio Costero de Recursos Acuáticos Calfuco, Campus Isla Teja, Valdivia, Chile; 2 Centro de Estudios Avanzados en Zonas Aridas (CEAZA), Facultad de Ciencias de Mar, Universidad Católica del Norte, Larrondo 1281, Coquimbo, Chile; National Institute of Water & Atmospheric Research, New Zealand

## Abstract

Environmental fluctuations can generate asynchronous species’ fluctuations and community stability, due to compensatory dynamics of species with different environmental tolerances. We tested this hypothesis in intertidal hard-bottom communities of north-central Chile, where a persistent upwelling centre maintains a mosaic in sea surface temperatures (SST) over 10s of kilometres along the shore. Coastal upwelling implies colder and temporally more stable SST relative to downstream sites. Uni- and multivariate analyses of multiyear timeseries of SST and species abundances showed more asynchronous fluctuations and higher stability in sites characterised by warmer and more variable SST. Nevertheless, these effects were weakened after including data obtained in sites affected by less persistent upwelling centres. Further, dominant species were more stable in sites exposed to high SST variability. The strength of other processes that can influence community stability, chiefly statistical averaging and overyielding, did not vary significantly between SST regimes. Our results provide observational evidence supporting the idea that exogenously driven compensatory dynamics and the stabilising effects of dominant species can determine the stability of ecosystems facing environmental fluctuations.

## Introduction

Forecasted scenarios of species loss, ecological phase shifts, and global environmental change have fuelled extensive research on community stability [1], [2]. Today, we recognise that community stability, measured as the temporal change in ecosystem properties like community productivity and abundance, depends on the strength of at least four mechanisms: compensatory dynamics, species dominance, statistical averaging, and overyielding [1], [3], [4]. Stability varies widely among natural communities [5] and it still is unclear how stabilising mechanisms interplay across different environmental conditions.

The stabilising mechanisms listed above depend on the magnitude and type of temporal variation in species abundances. For example, asynchronous and compensatory dynamics of competing species with differential environmental tolerances can maintain stability, because the decrease of stress-intolerant species can be compensated by growth of others (reviewed in [6]). Recent studies show that dominance may lead to more stable assemblages [7], as the abundance of competitively superior species can be less variable (e.g. resistant to disturbance) than subordinate ones [8]. Dominance, therefore, may increase stability by means of a mechanism analogous to the sampling effect [7], [9]. In addition, recent theoretical work indicates that unevenness in species abundances influences both, species synchrony and the average population variability [10]. Statistical averaging is another mechanism responsible for community stability. Since variance is a power function of the mean [11], species with large abundances display exponentially larger population variance than species with small abundances. The slope of the mean-variance scaling relationship, *z*, determines the contribution of statistical averaging to stability, with 1<*z*<2 meaning that increasing diversity increases and dampens population- and community-level variability, respectively [4], [12]. Finally, overyielding enhances stability and takes place when mean community-level properties, such as total abundance, increases with diversity faster than its variance [13]. Statistical averaging and overyielding are not mere artefacts. For example, *z* values <2 and disproportional increases of the temporal mean of an aggregate property can be caused by fundamental ecological mechanisms such as negative species interactions and resource complementarity, respectively [13], [14].

Theory and laboratory-based experiments indicate that variations in environmental factors can affect the strength of mechanisms promoting stability, particularly compensatory dynamics (e.g. [15]–[17]). Large environmental fluctuations in abiotic factors like temperature can generate synchronous fluctuations and reduce stability [18]. However, if different species are competitively superior at different times, following differential environmental tolerances, and if the effects of competition and environmental stress on per capita growth rates are positively correlated, environmental variability can actually lead to asynchronous dynamics in species fluctuations [19]–[21]. These exogenously driven compensatory dynamics (sensu [sensu 6]) can have strong stabilising effects on aggregate ecosystem properties [20].

In coastal ecosystems, variations in oceanographic and atmospheric conditions represent a relevant source of environmental fluctuations for coastal communities. Along mid-latitude, eastern oceanic boundaries, earth’s rotation, and prevailing equatorward winds displace coastal surface waters offshore, which are replaced by cold, nutrient-rich water upwelled from the subsurface. The process of coastal upwelling–usually locked to coastal promontories or headlands and where upwelling-favourable winds maintain nearshore waters cold–drives a strong temperature inversion at the ocean-atmosphere interface and maintains a shallow stratus cloud deck associated to onshore coastal topography [22], [23]. As a consequence, bays located downwind from upwelling centres show different temporal patterns of variation in sea surface temperature (SST), which are evidenced in the temporal structure of SST variance (e.g. [24]). A growing body of evidence indicates that such persistence in spatially structured variation in oceanographic conditions over 10's to 100's of km (i.e. mesoscale) is correlated to spatial variation in species- and community-level properties. In intertidal habitats, the temporal stability in body temperatures of mussels [25] and growth rates of corticated algae [26], [27] are positively affected by upwelling activity [28]. Moreover, recruitment of intertidal sessile invertebrates, in addition to positive biotic interactions, seems to be dampened [26], [28], [29]. Accordingly, the structure of sessile hard-bottom assemblages appears to be significantly correlated to mesoscale regimes of SST variance [24], [30]. If temporal variation in environmental conditions affects temporal variation in species abundance, then it can be predicted that temporal SST patterns will be associated to the strength of compensatory dynamics and stability of intertidal communities.

Hereby, we tested the general hypothesis that environmental fluctuations lead to compensatory dynamics and stability in intertidal hard-bottom communities. Our model system was the north-central coast of Chile (ca. 30°S), where a persistent upwelling centre, extending ∼100 km, maintains significant mesoscale differences in climatic and oceanographic conditions [22]. We tested the specific predictions that (1) sites affected by persistent and reduced upwelling activity show different patterns of temporal variation in SST, and (2) these two environmental regimes drive significant differences in species synchrony and community stability of hard-bottom communities. We further tested whether (3) the strength of other stabilising mechanisms, such as species dominance, statistical averaging, and overyielding, covary with the temporal variation in SST.

## Materials and Methods

This study was conducted as part of the activities carried out by the Changolab at the Centro de Estudios Avanzados en Zonas Áridas (CEAZA) and approved by its bioethics committee. In addition, the study conforms to the Art. §7 Law 19.300 “Bases Generales del Medio Ambiente”, which supports research on ecology and environmental science. No specific permits were required for the described field studies, because they were based on non-destructive estimations of species abundances and only few specimens (mostly filamentous algae) were collected when in situ identification was unfeasible. These species were not among endangered or protected species and locations were not privately-owned or protected by law.

### Study Sites

**Figure 1:**
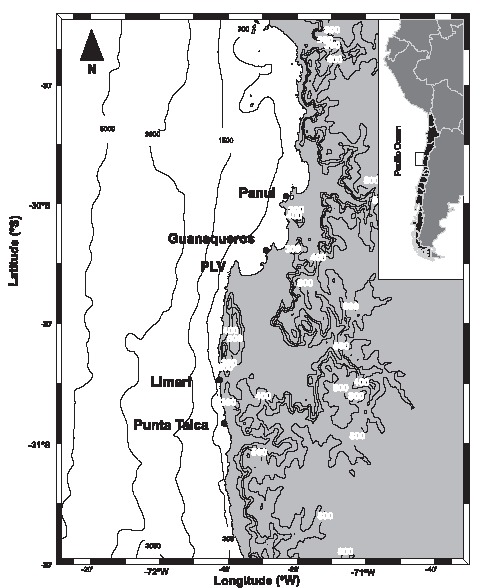
Location of the four sampling sites (Panul, Guanaqueros, Limarí, and Punta Talca) around the persistent upwelling centre located south Punta Lengua de Vaca (PLV). Bathymetric and topographic reliefs are also shown.

Observations were conducted on the north-central rocky shores of Chile, around a persistent upwelling centre (Fig. 1). Punta Lengua de Vaca, around 30.5°S (PLV hereafter), is a 2.4 km long peninsula, extending towards the equator and abutting the southern end of a large bay system. Sites located south of PLV are characterized by persistent upwelling activity, while sites located inside the bay (i.e. north of PLV) may receive water upwelled at PLV, but they experience little wind-driven coastal upwelling [29]. The study area around PLV corresponds to the transition between two mesoscale regions with contrasting oceanographic conditions: a northern region characterised by low eddy activity driven by weak but sustained upwelling-favourable winds, and a southern region characterised by high eddy activity driven by strong and prolonged episodes of upwelling favourable winds [31]–[34]. Accordingly, events of relaxation from upwelling are more frequent south than north of PLV [29]. Episodes of sea surface warming after relaxation from upwelling dominate high-frequency variability in SST, particularly during spring [32]. Such warm events can play a key biological role as onshore advection of recently upwelled waters can bring planktonic larvae and high nutrient concentrations to the intertidal community [35] and can lead to phytoplankton blooms nearshore [36].

We selected four sites that differed in their position relative to the PLV upwelling centre (Fig. 1). The southern sites, Limarí and Punta Talca, were located near the core of the upwelling centre; the northern sites, Panul and Guanaqueros, were located inside the large bay abutted by PLV. Southern and northern sites were, therefore, exposed to high and low intensity of upwelling activity, respectively.

The study region is characterised by spatial variation in terms of composition and abundance of species. For example, the dominant corticated red alga *Mazzaella laminarioides* and the large bull-kelp *Durvillaea antarctica* find their northern geographic range limit around this zone [37]. In addition, beds of the purple mussel *Perumytilus purpuratus,* characteristic on the mid intertidal zone of south-central shores, become scarce and are replaced by increased empty space and patches of chthamalid barnacles [29]. Despite these changes, opportunistic algae like *Ulva compressa*, *U. rigida*, and *Pyropia* sp. are persistent across the entire study region [37]. In the high and mid-intertidal zones, mobile grazers include several species of scurrinid limpets (*Scurria* spp.), chitons, and keyhole limpets (e.g. *Fissurella crassa*). In the low intertidal zone, the kelp *Lessonia nigrescens* and the bull kelp *Durvillaea antarctica* form dense canopies and support a diverse assemblage of mobile invertebrates, including chitons like *Enoplochiton niger* and *Chiton granosus,* keyhole limpets like *Fissurella limbata,* and turban snails like *Tegula* spp. and *Prisogaster niger* [38]–[40].

### Sampling

At each site, we selected mid-intertidal rocky platforms with similar slopes, wave-exposure, and orientation to prevailing southwest winds, where we estimated species percentage cover on ten 0.25 m^2^ plots located along ca. 20 m alongshore transects. The position of each plot was permanently marked with stainless-steel bolts and rings. Plots were haphazardly positioned, but positions were restricted to flat and gently sloped surfaces lacking crevices and tide pools. This protocol has been used in several studies on benthic diversity along Chilean coasts and elsewhere (e.g. [5], [37], [38], [41]).

Percentage cover of each sessile macro-epibenthic (>5 cm) species was estimated on each plot every three months between May 2009 and February 2012. Estimations were conducted during daytime low-tide intervals and to the nearest 1% by means of 0.25 m^2^ quadrats subdivided in 25 equal fields. Percentage cover directly reflects resource availability in hard-bottom communities, where competition for settlement surfaces is a pivotal driver of species dynamics [42]. In addition, percentage cover is routinely used as a proxy for species abundances [43], and can be significantly correlated with biomass on these shores [24] and elsewhere [44], [45]. Accordingly, we assumed that percentage cover was appropriate to assess temporal dynamics in species abundances.

Our study represented a conservative estimate of the biodiversity at each site, because observations were focused on macro-epibenthic organisms for which appropriate taxonomic keys were available and therefore were possible to accurately be identified in the field [46]–[48]. Nevertheless, the resulting subset of species encompassed a diverse taxonomic group that included several phyla and species from at least three trophic levels. Organisms were identified in the field to the lowest possible taxonomic level and few specimens were collected from adjacent areas and identified in the laboratory. We considered that a mixture of taxonomic resolution was appropriate for assessing community-level stability, as previous work shows that similar patterns in community structure are apparent whether fine, coarse, or mixed taxonomic resolution is used [49], [50]. Further details of sampling procedure in these sites are published elsewhere [37], [38].

We used *in situ* measurements of sea surface temperature (SST) to characterise the environmental variability. Between August 2009 and August 2011, we deployed at each site temperature data-loggers that recorded SST every 10 minutes. Each data-logger was enclosed into a PVC pipe, which was inserted into a concrete block moored at 1-m depth [51].

### Statistical Analyses

The magnitude of environmental fluctuations in each site was estimated using temporal variances calculated from the two-year daily SST measurements where the few missing observations in the time series were interpolated using a cubic spline. Temporal variances were calculated separately from daily to trimonthly temporal scales (1, 5, 30, and 90 days) using a running-mean filter.

In order to illustrate multivariate patterns in community structure across sites, we used canonical analysis on principal coordinates (CAP [52]). CAP is a constrained multivariate method that uses an a priori hypothesis to produce an ordination plot, allowing the detection of patterns that could be masked by overall dispersion in unconstrained methods such as multidimensional scaling. CAP plots were based on a fit between a vector of sites and a matrix of Bray-Curtis dissimilarities calculated from raw percentage cover data. In addition, individual taxa that might have influenced any differences among sites seen in the CAP ordination were investigated by calculating product-moment correlations of species abundances with canonical ordination axes. We included in CAP only taxa having a mean percentage cover >5% in order to reduce distortion in the ordination plots. The test for different levels of stability between sites was complemented in a multivariate context by means of a permutation distance-based test for homogeneity in multivariate dispersion (i.e. PERMDISP [53]). The significance of F-statistic from ANOVA of Bray-Curtis distances to group centroids was interpreted by means of 999 permutations of model residuals that generated a distribution of F under the null hypothesis of no difference in dispersion between sites.

Stability has several meanings in ecology, which include concepts like the magnitude of disturbances a system can tolerate (domain of attraction), the magnitude of changes driven by a disturbance (resistance), how long a the system requires to return to a specified fraction of its initial state (resilience) and how much a measure varies over time (variability) [54]–[57]. Pioneer studies on ecological stability explicitly considered stability as related to temporal variation in ecosystem properties [58], [59]. In this work, we used the temporal variability in total community cover as an overall measure of stability. Community stability was expressed as the ratio between the long-term temporal mean and standard deviation (*S = μ/σ*) of total percentage cover; i.e., the sum of the abundances of all species in the assemblage [13]. Both, *μ* and *σ* were formally analysed to address whether mesoscale variation of stability was related to variations of mean abundances or temporal variability.

In order to measure community-wide synchrony in species abundances, we used the *φ_x_* statistic [60]:
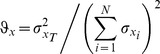
(1)where *φ_x_* describes the variance in the sum of all species abundances (i.e. total community abundance, *x_T_*) relative to the squared sum of the standard deviations of all *N* individual species (*x_i_*). *φ_x_* is standardised between 0 (perfect asynchrony) and 1 (perfect synchrony; i.e. most species are positively correlated); and is independent of the magnitude and distribution of species abundances and variances, allowing quantitative comparisons of communities with different species richness. The *φ_x_* statistic uses the fact that the variance of an aggregate property can be partitioned into the sum of all species variances plus the sum of all pair-wise species covariances [61] such that:



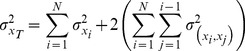
(2)Species dominance was expressed as 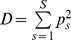, where *D* is the Simpson dominance index, 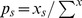, *x* is the relative abundance of the *s*th species, and *S* is the number of species in the sample. Statistical averaging effects depend on how the temporal variance in the abundance of a species changes with its temporal mean [12]. The scaling of variance *σ* ^2^ with the mean *μ* is described by Taylor’s power function, *σ* ^2^ = *c µ^z^,* where *c* is a constant and *z* is the scaling coefficient [11]. The value of *z* affects the strength of the statistical averaging, with 1<*z*<2 meaning that diversity increases and dampens the population- and community-level variability, respectively [12]. The logarithmic transformation of *σ* ^2^ = *c µ^z^* results in a linear equation in the form of log (*σ* ^2^) = *c*+*z* log (*μ*). We fitted this linear relationship to the entire dataset, combining all species. The *z*-values obtained from each regression were used as dependent variables. In addition, regression residuals for each species were used to estimate species-level stability, and thus infer whether species with larger percentage covers (i.e. dominant species) were more stable; larger negative residuals indicated higher species-level stability [7]. We tested the relationship between species-level stability and dominance by estimating Pearson product-moment correlation coefficients (*r*) between residuals and the long-term means of species’ percentage covers. Significance was tested for each site by testing observed correlation coefficients against a null distribution of *r* coefficients generated from 10000 randomisations [7]. Overyielding was estimated for each plot as the slope of the regression of total community cover vs. taxon richness [13]. Within each plot, the regression was calculated with the sampling dates as replicates and the effect of temporal autocorrelation was reduced by means of a mixed-effects model and best linear unbiased predictors [5].

We used separate 2-way nested analyses of variance (ANOVAs) with ‘region’ (2 levels: either north or south PLV) as a fixed factor and ‘site’ (2 levels) as a random factor and nested in ‘region’ to test the prediction that sites with different degrees of temporal variation in SST show significant differences in stability and the stabilising mechanisms. After running the full models, highly conservative (*P*≥0.25) error terms were removed and F-ratio statistics were then recalculated with a pooled denominator [62]. Homogeneity of variance was graphically explored by means of residuals-vs.-fits and normal Q-Q plots. In order to achieve homogeneity of variances, data of *S* and *φ_x_* were log_10_ transformed, and those of *σ* were square root transformed.

In addition to the datasets described above, we analysed a multiyear (1998–2005) dataset of species percentage covers estimated in 17 sites and spanning ca. 733 km of the coast (29.47°S–36.07°S). In each of these sites, species percentage covers were estimated on randomly located 0.25 m^2^ plots on a regular basis. Most sites were sampled every six months from 1998 to 2000 and then from 2003 to 2005 (see Table S1). Each site was categorised either as exposed to warm or cold SST according to Broitman et al. [38], Nielsen and Navarrete [27], and Wieters [26]. For each site, we estimated temporal variance components in total community cover (*σ^2^*) by means of restricted maximum likelihood (REML). The regional trend in variance components was removed after calculating the residuals from locally weighted regression scatterplot smoothing (LOWESS) against latitude. The hypothesis that stability (i.e. the inverse of temporal variability in total community cover) differs between sites exposed to warmer and colder SST was tested with a 1-way ANOVA with ‘SST’ (two levels, cold and warm SST) as fixed factor and the residuals of variance components as dependent variable. SST analyses were conducted in Matlab (v7.8.0, The Mathworks, Natick, Massachusetts, USA). All other estimations and statistical analyses were conducted in R 2.15.0 [63].

## Results

**Figure 2:**
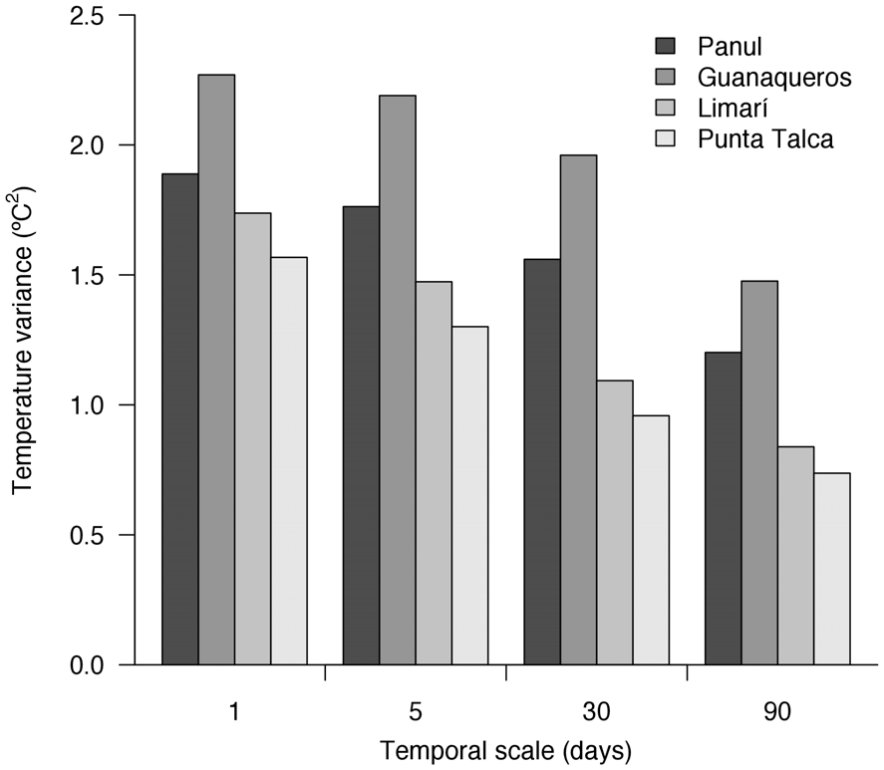
Long-term variance in sea surface temperatures (SST) recorded in sites located north (Panul and Guanaqueros) and south (Limarí and Punta Talca) the upwelling centre at Punta Lengua de Vaca (PLV). SST values were recorded every 10 minutes at 1 metre depth.

Our observations showed that SST was higher at Panul (median = 13.7°C, range = 12.4–17.4°C) and Guanaqueros (14.1°C, 12.3–17.9°C), both sites located north of Punta Lengua de Vaca (PLV); than at Limarí (12.7°C, 11.3–16.7°C) and Punta Talca (12.6°C, 11.2–16.5°C), both located south of PLV. Temporal variances of SST were, on average, 50% larger at Panul and Guanaqueros that at Limarí and Punta Talca (Fig. 2). The differences between northern and southern sites were stronger when the variances were calculated at the monthly and trimonthly scales, where SST records were ca. 70% more variable north than south of PLV (Fig. 2). Across scales of variability, the largest and smallest variances were observed at Guanaqueros (2.23) and Punta Talca (0.74), respectively (Fig. 2). These results support the prediction that sites categorised as experiencing low (Panul and Guanaqueros, north of PLV) and high (Limarí and Punta Talca, south of PLV) upwelling activity show different patterns of temporal variation in SST.

**Figure 3:**
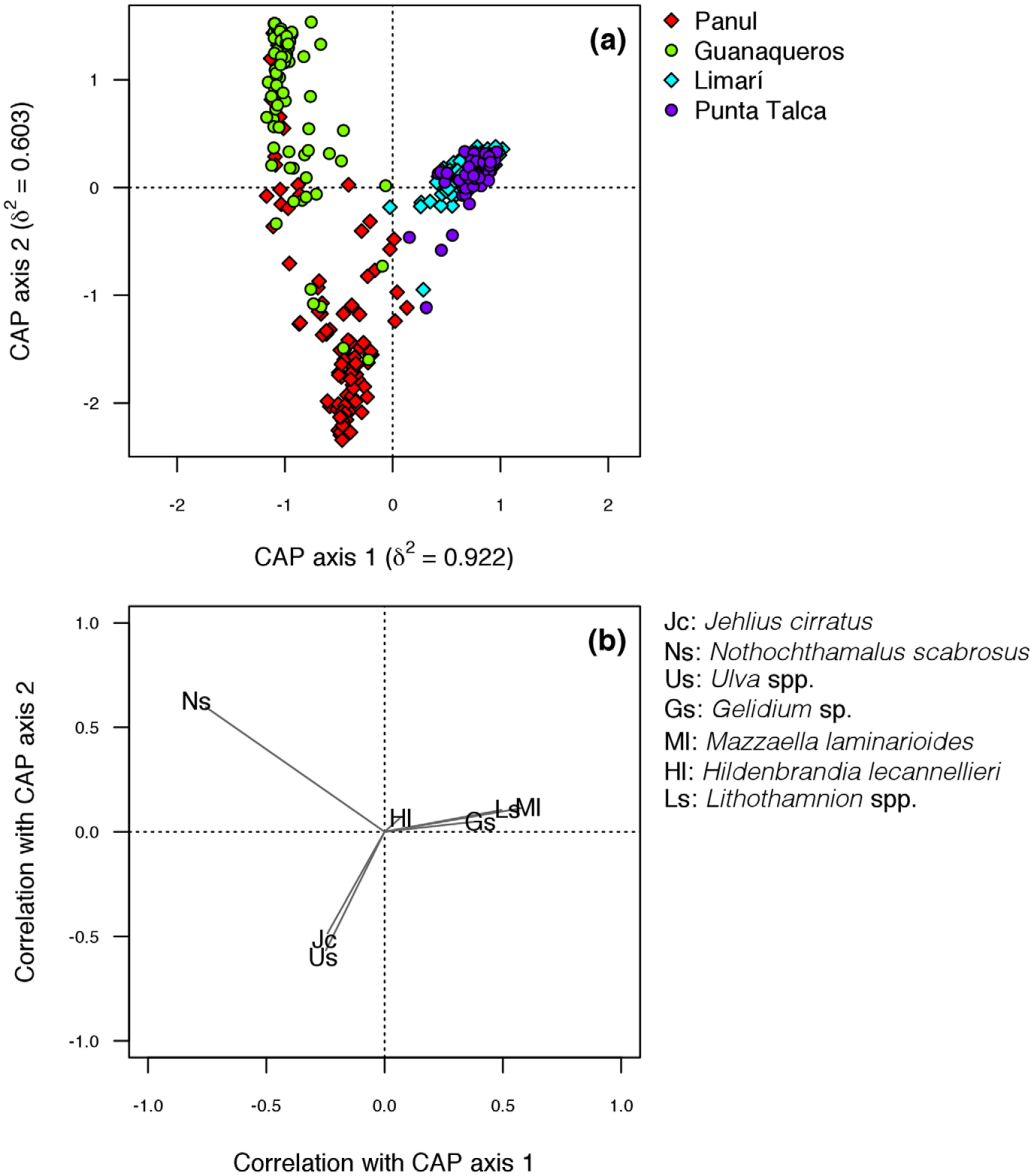
Canonical analysis of principal coordinates (CAP) ordination: (a) Canonical axes that best discriminate the assemblages from sites located north (Panul and Guanaqueros) and south (Limarí and Punta Talca) to the upwelling centre at Punta Lengua de Vaca (PLV). (b) Correlations of original taxa with canonical axes. CAP was significant with *P* = 0.005. Taxa with average cover <5% were excluded from the analysis in order to reduce distortion in the ordination.

According to the observed spatial structure in SST, sites north and south of PLV showed different community structures (Fig. 3). Canonical analyses of principal coordinates (CAP) showed larger multivariate dispersion in Guanaqueros and Panul (northern sites) than in Punta Talca and Limarí (southern sites, Fig. 3a). Permutation analyses of homogeneity of group multivariate dispersion showed significant differences among sites (pseudo-F_3, 396_ = 60.059, P = 0.001). Pairwise comparisons and CAP ordinations (Fig. 3a) indicated that group-specific distances to centroids were Panul>Guanaqueros>Limarí = Punta Talca. Chthamalid barnacles dominated the assemblages north of PLV, while the corticated red alga *Mazzaella laminarioides* and the crustose *Lithothamnion* spp. dominated the sites south of PLV (Fig. 3b).

**Figure 4:**
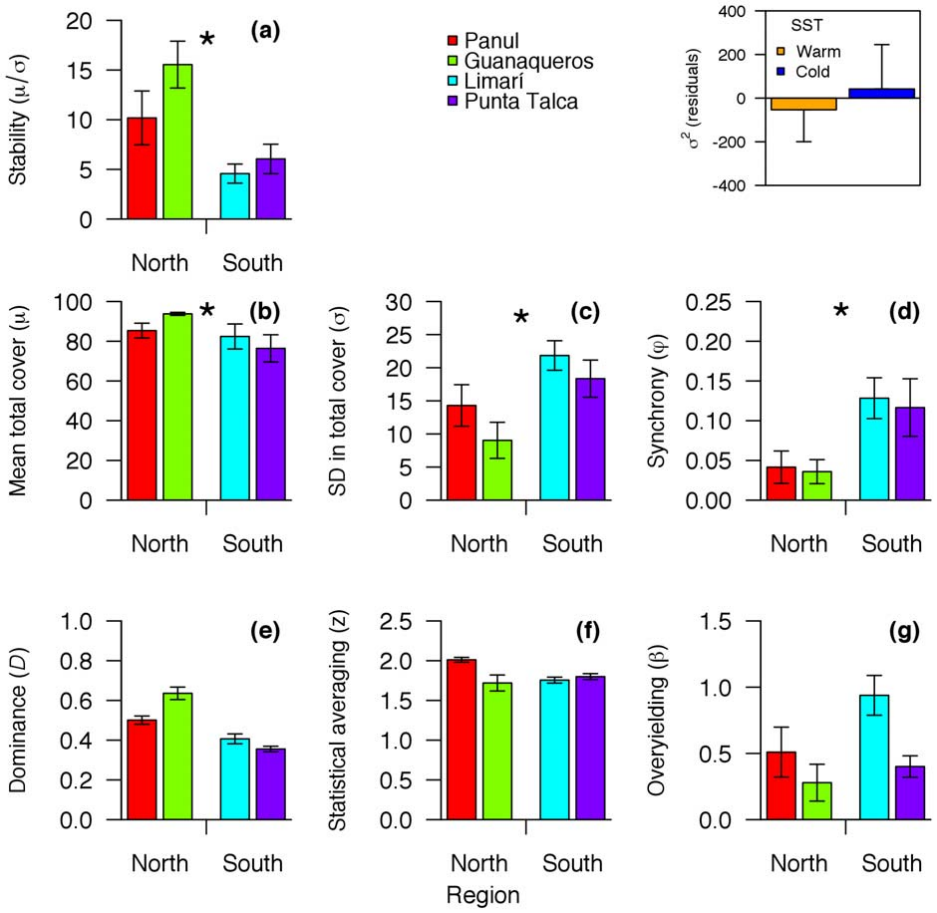
Temporal stability in sites located north (Panul and Guanaqueros) and south (Limarí and Punta Talca) to the upwelling centre at Punta Lengua de Vaca (PLV). (a) Stability measured as (b) long-term mean in total community cover divided by its (c) standard deviation. (d) Synchrony in species’ fluctuations. (e) Simpson’s index of dominance. (f) Statistical averaging of species’ fluctuations, expressed as the slope of the log_10_ transformation of Taylor’s power law. (g) Overyielding, measured as the slope of the regression between taxon richness and long-term mean in total community cover. (Insert) Temporal variance components of total community cover estimated by REML from 17 sites between 29.47°S and 36.07°S (see Table S1), after removing the regional trend in the data by means of LOWESS regression. Values are expressed as means ± SEM.

Community stability, measured as the ratio between the long-term mean and standard deviation in total community cover (*S* = *μ/σ*), was significantly higher at northern sites (Fig. 4a, Table 1). Accordingly, the analysis of regional residuals of the broad-scale dataset (1998–2005) indicated that warm sites showed lower variance components of total community cover than colder sites (insert in Fig. 4; mean residual variance components = −53.24 and 42.01 for warm and cold SST, respectively). These differences were however statistically insignificant (*F*_1, 15_ = 0.138, *P* = 0.715).

**Table 1:** Results of nested ANOVA of the effects of Region (north and south of Punta Lengua de Vaca, fixed factor) and Site (Panul, Guanaqueros, Limarí, or Punta Talca; random factor and nested within Region) on stability, species synchrony, species dominance, statistical averaging, and overyielding.

	Region (1)			Site(Region) (2)			Residual (36)	Pooled residual (38)	
Source of variation	MS	F		MS	F		MS	MS	MS_ denominator_
Response variable									
Stability	0.78	19.03	***	0.06	1.42		0.040	0.041	Pooled
Mean total cover	2284.63	8.27	**	166.40	0.59		282.455	276.347	Pooled
Std. dev. total cover	1009.08	13.03	**	144.22	1.96		73.733	77.442	Pooled
Species synchrony	3.54	21.82	***	0.19	1.16		0.161	0.162	Pooled
Dominance	0.43	8.73		0.05	8.32	**	0.006		Site(Region)
Statistical averaging	0.12	0.53		0.22	6.56	**	0.034		Site(Region)
Overyielding	0.76	0.89		0.85	4.10	**	19.930	19.056	Pooled

Degrees of freedom are in brackets. Error terms with highly conservative P-values (≥0.25) were removed from the model and the F-ratio for Region was recalculated with a pooled residual. Error terms used as denominator for each F-ratio are given in MS _denominator_.

** = *P*<0.05;

*** = *P*<0.001.

The long-term mean of total community cover (*μ*) was significantly larger north than south of PLV (Fig. 4b; Table 1). In addition, the long-term standard deviation in total community cover (*σ*) was significantly smaller at northern than southern sites (Fig. 4c, Table 1). Species synchrony was significantly lower north than south of PLV (Fig. 4d). Species dominance, statistical averaging, and overyielding did not vary statistically across the region (Figs. 4e, 4f, and 4g; Table 1).

**Figure 5:**
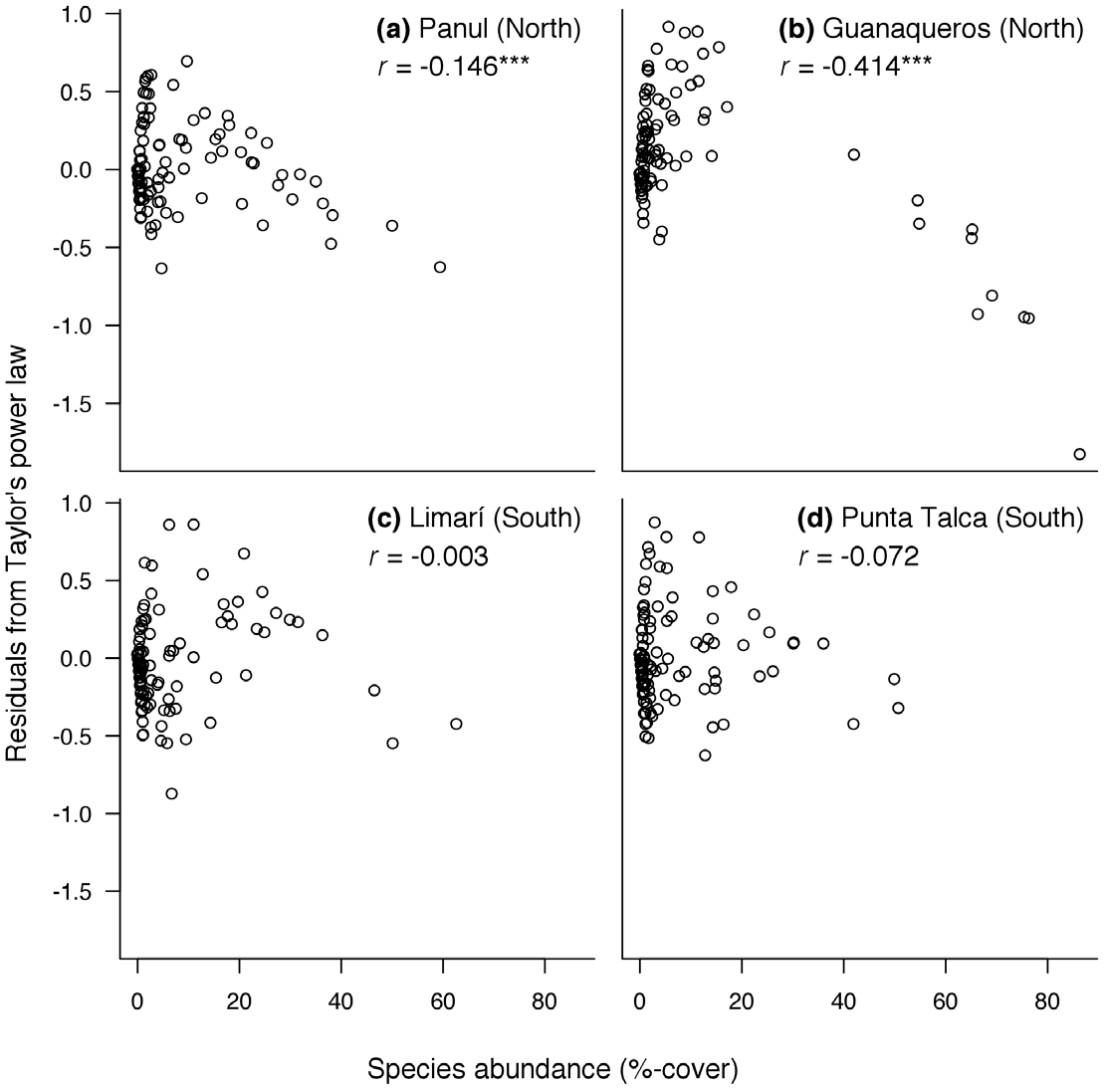
Correlation between species relative abundances (%-covers) and residuals from Taylor’s power law. (a, b) Sites located north and (c, d) south to the upwelling centre at Punta Lengua de Vaca (PLV). Larger negative residuals indicate more stable abundances.

Despite species dominance remained constant around PLV, the stabilising effects of dominant species probably differed between northern and southern sites. Significant negative relationships between Taylor’s power law residuals and relative abundances were observed only in Panul and Guanaqueros, both sites experiencing warm SST (Fig. 5, permutation correlation tests). Therefore, dominant species were more stable than subordinate species in sites north, but not south of, Punta Lengua de Vaca.

## Discussion

The mesoscale patterns here documented suggest that variation in environmental conditions over relatively small spatial scales can lead to significant differences in community-wide stability. We found that higher variability in sea surface temperature (SST) was related to higher stability and lower species synchrony (thus strengthened compensatory dynamics), but it was unrelated to other stabilising mechanisms such as statistical averaging and overyielding. Dominant species were significantly more stable than subordinate ones only in sites exposed to high SST variability, suggesting that particularly stable dominants may also have influenced community stability. Our findings agree with theoretical and empirical evidence for a positive effect of environmental fluctuations on compensatory dynamics. We suggest that ecological mechanisms evoking differential species’ responses to environmental fluctuations (e.g. storage effect [64], [65]) play a relevant role in generating compensatory dynamics and maintaining a constant provision of ecosystem properties under fluctuating environmental conditions.

Our analysis of SST showed that temperature records were warmer and more variable at sites north than south PLV, which experience reduced and intense upwelling activity, respectively. Despite the small differences in SST regimes detected, these confirm the difference in mesoscale oceanographic conditions across PLV. Our results are in good agreement with previous work showing significant differences between north and south PLV in terms of SST, CO_2_ saturation, and the magnitude and variability of wind speed [22], [32], [66], which can be associated with temporal fluctuations in primary productivity [36]. Accordingly, the spatiotemporal variation in SST is well correlated with that in biological oceanographic features and supports our interpretation of SST patterns as a proxy for local environmental conditions.

Following the stability metric we chose (*S* = *μ*/*σ,* [13]), higher values at sites experiencing warmer and variable SST indicated increased mean (*μ*) and decreased standard deviation (*σ*) of total community cover. The marginal difference in *μ* between upwelling regimes suggests that decreased temporal variability at sites affected by low upwelling intensity was more important. Species dominance, statistical averaging, and overyielding did not vary between environmental regimes, indicating that reduced synchronisation in species abundances can be associated to lower temporal variability [5] at sites experiencing high temporal variation in SST.

The differences in terms of stability and compensatory dynamics between environmental regimes can be explained in the light of differential amounts of temporal variability, assuming that climate and broad-scale processes strongly influence population synchrony [18], [67]. Separation of the temporal component of species’ niches promotes coexistence and compensatory dynamics [20]. Temporal changes in environmental factors like temperature stimulate compensatory dynamics thanks to differences in temporal resource use patterns between species [68], [69]. Long-term manipulative experiments suggest that environmental variation allows the expression of niche differences between competing species, which results in a positive effect of diversity on stability through niche partitioning [70], [71]. On the other hand, weakening of the diversity-stability relationship toward higher latitudes can be a response to the stronger influence of environmental factors on species richness and composition [72]. Thus, exogenous factors, such as environmental fluctuations, seem to have strong and determinant effects on the main mechanisms driving community stability.

The effects of dominant species did vary between warm and cold sites. Only in the northern sites, residuals from Taylor’s power law estimated from dominant species were significantly lower than those estimated from subordinate species. This indicates that dominant species were less variable over time in sites exposed to warm and variable SST, and that such dominant species may have influenced the community-wide stability north Punta Lengua de Vaca. These communities were dominated by chthamalid barnacles, which have a complex life cycles with a planktonic stage that might last for several weeks, allowing barnacles to rapidly colonise disturbance-generated patches of empty substratum [73]. It might be hypothesised that increased supply of barnacle larvae to our low-upwelling study sites [29] might have conferred stability to the whole community by re-colonising patches of bare rock generated by disturbances or predation (e.g. by the sea sun *Heliaster helianthus*; [74]). From a metacommunity perspective, the mass effect of immigration from source populations can be seen as a mechanism that buffers population abundances when environmental conditions are unfavourable for a given species [75]. In addition to differential environmental responses, mass effects are pivotal for stability at the ecosystem-level [16]. Accordingly, the storage effect model [64] seems to fit to our observational data, given the dominance of long-distance dispersing species in sites with stronger compensatory dynamics.

Following the negative relation between recruitment of chthamalid barnacles and upwelling intensity [30], [76], barnacles were sparse south PLV and the assemblages were dominated by red algae, which usually experience strong dispersal limitation [77]. Therefore, it can be suggested that local mechanisms are relevant in driving the structure and stability of these communities. One type of these local processes might correspond to facilitative effects on other species. For example, *Mazzaella laminarioides*’ leathery morphology can generate understorey environmental conditions different from the overall environment, and thus positive covariance among obligate understorey species as seen in other temperate shores [5], [78], [79]. In central Chile, the abundance of crustose algae like *Lithothamnion* spp. are positively correlated with that of corticated algae [38], suggesting that crustose species, which are specialised to shaded conditions [80], can colonise more stressful habitats thanks to habitat amelioration by the dominant *M. laminarioides*. These positive relationships might have contributed with synchronous species dynamics and thus with lower stability in both sites south PLV. Upwelling intensity, however, can dampen positive interactions, probably due to enhanced nutrient concentrations. Manipulative evidence shows that the turf-forming alga *Gelidium chilense* has stronger facilitative effects on recruitment of *P. purpuratus* on areas downstream of upwelling sites [26]. The role of morphological traits of habitat-forming species should be comparatively analysed in order to assess the effect of positive interactions on species synchrony across differential environmental settings.

Here, we have presented multiyear observational evidence for a relationship between environmental fluctuations and community stability. We have observed contrasting structural configurations in low- vs. high-upwelling sites, defined by life histories that respond to different scales of environmental variation: life-histories that respond to broad-scale processes (i.e. dispersal) dominated low-upwelling sites, while life-histories that respond to fine-scale environmental variation dominated high-upwelling sites. Our results indicate that higher environmental variability might lead to stronger compensatory dynamics and thus to higher community-wide stability. Such a relationship has been largely proposed by theoretical models and laboratory-based experiments, but rarely by field observations that integrate natural filters and broader environmental conditions. Still is necessary to test for differences in environmental tolerances between sites experiencing different levels of environmental fluctuations; manipulative experiments seem ideal to test if this assumption holds at the mesoscale. Our results support the idea that exogenously driven compensatory dynamics, in addition to stabilising effects of dominant species, can determine the stability of ecosystems facing environmental fluctuations.

## Supporting Information

Table S1 **Summary of sampling sites used to estimate variance components (REML) of total community cover**. Species percentage covers were estimated seasonally in each site from 1998 to 2005 (Sampling year). *Sites were categorised as experiencing warm or cold sea surface temperatures (SST), according to satellite imaging and in situ records [1]–[3].
(DOC)
