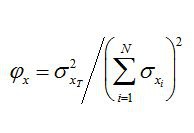# Correction: Mesoscale Variation of Mechanisms Contributing to Stability in Rocky Shore Communities

**DOI:** 10.1371/annotation/e47a789f-3e44-46b0-b9eb-198277781141

**Published:** 2013-10-10

**Authors:** Nelson Valdivia, Andrés E. González, Tatiana Manzur, Bernardo R. Broitman

The symbol on the left side of equation 1 should be a "phi". Please see the correct Equation 1 here: