# Supplementary material for: Mesoscale Variation of Mechanisms Contributing to Stability in Rocky Shore Communities
Source: PLoS One. 2013 Jan 11;8(1):e54159. doi: 10.1371/journal.pone.0054159 (PMC3543366; doi:10.1371/journal.pone.0054159)
Supplement: Table S1 — Summary of sampling sites used to estimate variance components (REML) of total community cover. Species percentage covers were estimated seasonally in each site from 1998 to 2005 (Sampling year). *Sites were categorised as experiencing warm or cold sea surface temperatures (SST), according to satellite imaging and in situ records [1]–[3]. (DOC) [file pone.0054159.s001.doc]

| Site | Latitude | Longitude | SST* | Sampling year |
| --- | --- | --- | --- | --- |
|  |  |  |  |  |
| Temblador | -29.47 | -71.32 | Warm | 98-00-03-04-05 |
| Arrayán | -29.69 | -71.32 | Warm | 98-00-04-05 |
| Guanaqueros | -30.20 | -71.47 | Warm | 98-99-00-03-04-05 |
| Punta de Talca | -30.93 | -71.50 | Cold | 98-99-00-03-04-05 |
| Puerto Oscuro | -31.41 | -71.61 | Warm | 98-00-03-04-05 |
| Los Molles | -32.23 | -71.50 | Warm | 98-00-03-04-05 |
| Curaimilla | -33.10 | -71.73 | Cold | 98-03-04-05 |
| Quintay | -33.18 | -71.70 | Cold | 98-99-00-03-04-05 |
| Quisco | -33.38 | -71.70 | Warm | 98-99-00-03-04-05 |
| ECIM-Marine reserve | -33.50 | -71.63 | Warm | 98-99-03-04-05 |
| Las Cruces | -33.51 | -71.63 | Warm | 98-99-03-04-05 |
| Matanzas | -33.96 | -71.88 | Cold | 98-99-03-04-05 |
| Punta de Lobos | -34.43 | -72.05 | Cold | 98-99-00-03-04-05 |
| Bucalemu | -34.65 | -72.05 | Cold | 98-00-04-05 |
| Constitución | -35.35 | -72.45 | Cold | 98-99-00-03-04-05 |
| Pelluhue | -35.82 | -72.60 | Cold | 98-99-03-04-05 |
| Buchupureo | -36.07 | -72.80 | Cold | 98-99-00-03-04-05 |

**References**

1. Broitman BR, Navarrete SA, Smith F, D. GS (2001) Geographic variation of southeastern Pacific intertidal communities. Mar Ecol Prog Ser 224: 21-34.

2. Nielsen KJ, Navarrete SA (2004) Mesoscale regulation comes from the bottom-up: intertidal interactions between consumers and upwelling. Ecol Lett 7: 31–41.

3. Wieters EA (2005) Upwelling control of positive interactions over mesoscales: a new link between bottom-up and top-down processes on rocky shores. Mar Ecol Prog Ser 301: 43-54.
